# Saliva Sampling Material Matters: Effects on the Results of Saliva Analysis in Pigs

**DOI:** 10.3390/ani13243757

**Published:** 2023-12-05

**Authors:** Alba Ortín-Bustillo, María Botía, Marina López-Arjona, Luis Pardo-Marín, José J. Cerón, Silvia Martínez-Subiela, María José López-Martínez, Asta Tvarijonaviciute, Alberto Muñoz-Prieto, Camila P. Rubio, Silvia Martínez-Miró, Damián Escribano, Fernando Tecles

**Affiliations:** 1 Interdisciplinary Laboratory of Clinical Analysis of the University of Murcia (Interlab-UMU), Regional Campus of International Excellence ‘Campus Mare Nostrum’, University of Murcia, Campus de Espinardo s/n, 30100 Murcia, Spain; alba.ortinb@um.es (A.O.-B.); maria.botiag@um.es (M.B.); lpm1@um.es (L.P.-M.); jjceron@um.es (J.J.C.); silviams@um.es (S.M.-S.); mariajose.lopez28@um.es (M.J.L.-M.); asta@um.es (A.T.); alberto.munoz@um.es (A.M.-P.); camila.peres@um.es (C.P.R.); ftecles@um.es (F.T.); 2 Department of Animal and Food Science, School of Veterinary Science, Universitat Autònoma de Barcelona, 08193 Cerdanyola del Vallès, Spain; marina.lopez.arjona@uab.cat; 3 Department of Animal Production, Regional Campus of International Excellence ‘Campus Mare Nostrum’, University of Murcia, Campus de Espinardo s/n, 30100 Murcia, Spain; silviamm@um.es

**Keywords:** analytes, collection material, pig, stimulated saliva

## Abstract

**Simple Summary:**

There is an increasing interest in measuring biomarkers from saliva, as samples can be obtained noninvasively. This is particularly true in pigs where blood sampling is technically complicated and stressful. In pigs, studies regarding how the absorbent material used for collection affects analytical results are scarce. The aim of this study was to evaluate how the material used for saliva collection can affect the results of analyses for the different types of biomarkers that can be measured in the saliva of pigs. In this report, polypropylene sponges gave higher sample saliva volumes and, in some salivary analytes, provided different values compared with cotton rolls. Therefore, it can be concluded that the type of material used can influence the results of saliva analysis.

**Abstract:**

The use of saliva as a biological sample from pigs is of high practical interest because blood collection from pigs is difficult and stressful. In this study, the influence of two different materials, a cotton roll and a polypropylene sponge, in porcine saliva collection was evaluated. For this purpose, the effect of the material used for sampling was evaluated in a panel of 13 analytes, including those related to stress (cortisol and oxytocin), inflammation and immunity (adenosine deaminase, haptoglobin and myeloperoxidase), redox homeostasis (the cupric reducing ability of saliva, the ferric reducing activity of saliva, and the Trolox equivalent antioxidant capacity), and sepsis (procalcitonin), as well as other routine analytes related to metabolism and different tissues and organs, such as lactate dehydrogenase, creatine kinase, urea, and total protein concentration. The polypropylene sponge provided a higher sample volume than the cotton roll. Although the results of some salivary analytes were equivalent for both materials, other analytes, such as creatine kinase, haptoglobin and total proteins, showed significant differences depending on the material used for saliva collection. Therefore, the type of material used for salivary collection in pigs should be considered when interpreting the results of analyses of the salivary analytes.

## 1. Introduction

Saliva has been increasingly used in recent years as a biological sample because it can be obtained by non-invasive procedures, avoiding the pain and disturbance to the individuals associated with blood sampling [1,2]. In addition, the procedure for collection is easy and there is no need for specialized staff because collection can be performed by nontrained personnel [3]. All these advantages are especially relevant in pigs because blood collection can be highly stressful when animal restraint is needed [4]. Traditionally, porcine saliva has been used for measuring cortisol for stress evaluation [4,5] and the detection of infectious diseases [6]. However, many other biomarkers related to diverse pathophysiological situations, such as immunity, redox homeostasis, and inflammation, can also be measured in pigs [7]. This makes saliva a convenient source of biological samples for this species.

In humans, saliva can be collected by passive drool or by different absorbent materials such as cotton, polyethylene, polypropylene or polyester. Cotton is among the most commonly used materials for saliva collection in humans, but several studies indicate that it could affect the analytical results of the salivary biomarkers compared with unstimulated saliva. A previous report showed that saliva collected by passive drool had differences in protein components analysed by proteomics compared to cotton [8]. In addition, cortisol [9,10,11], C-reactive protein [12] or melatonin [13] were lower in saliva collected by cotton compared with passive drool. However, some controversy has been found in the case of cortisol, because a recent study did not find any effect of cotton on cortisol measurement [14]. This divergence could be related to a different method used for cortisol measurement. On the other hand, higher levels of testosterone and estradiol have been reported when saliva is collected with cotton [15]. In addition to this, changes can also occur when other absorbent materials are used. For instance, polyethylene has provided lower values for testosterone and androstenedione concentrations [14]. Furthermore, polypropylene covered polyether rolls have provided higher concentrations of thiocyanate and IgA and lower lysozyme activity than saliva obtained by stimulation with paraffin wax [16]. In addition, polyester and polyethylene devices have produced significant increases in salivary levels of leptin and insulin compared to cotton, whereas polyester has provided higher amylase activity and similar IgA concentration to cotton [17]. All these results point to the influence that the material used in saliva collection can have on the analytical results. The underlying mechanism of how a material can affect the results is unknown. It has been postulated that constituents of the material could interact with the antibodies used in immunological methods, or that some traces of saliva could be retained in the materials, affecting mainly those analytes with very low concentrations [15]. Moreover, the use of materials usually implies the use of sample centrifugation as opposed to passive drooling, and this centrifugation could affect the results [8].

In pigs, the saliva is usually obtained using an absorbent material because the collection by passive drooling is complex and difficult. To the authors’ knowledge, studies in which different saliva collection systems were evaluated in this species are scarce. In one report, saliva was obtained using cotton, hemp or nylon ropes and used for the detection of the porcine reproductive and respiratory syndrome virus (PRRSV) by serology (IgG, IgM or IgA anti-PRRSV by ELISA) or reverse transcription polymerase chain reaction (qRT-PCR). The results showed that the type of material used to collect saliva did not have a significant impact on the results of IgG-based PRRSV antibody ELISA and total IgG assay. However, nylon provided the highest values of total IgM and IgA, whereas cotton provided the highest results in the PRRSV qRT-PCR assay [18]. In a recent report, two methods (cotton ropes and polypropylene sponges) that can be used to collect porcine saliva for biomarker analysis were evaluated. Rope-collected saliva provided higher values than sponges for analytes such as cortisol among others, although this effect was attributed to the higher degree of dirtiness obtained with ropes [19]. In spite of the importance that saliva analyses in pigs are gaining, no other studies have been found regarding the effect of the saliva collection material on this species.

The hypothesis of this research is that the use of different collection materials in saliva sampling could differently affect the levels of salivary biomarkers, and that this knowledge would be of interest for a better interpretation of the results. The aim of this report was to study the influence that the use of two different materials (cotton and polypropylene) for saliva collection in pigs could have on the results of analyses for different salivary biomarkers. For this purpose, saliva was collected from pigs by using two different materials: a cotton roll and a polypropylene sponge. Those two materials were used because, according to the authors’ knowledge, they are among the most frequently used for saliva collection in pigs. In addition, both healthy pigs and also pigs with lameness were sampled in order to evaluate the influence of the collection material at different concentrations of analytes because previous studies have shown that many of the biomarkers included in this study can change in pigs with lameness [20]. In this preliminary report, a panel of analytes was selected in order to have at least one representative analyte of each group of biomarkers that can be used in saliva, namely stress and welfare, such as cortisol and oxytocin (which is considered as a marker of positive welfare according to previous reports [21]); inflammation and immunity, such as adenosine deaminase (ADA), haptoglobin (Hp) and myeloperoxidase (Mpx); sepsis, such as procalcitonin (PCT); and redox homeostasis, such as the cupric reducing ability of saliva (CUPRAC), the ferric reducing activity of saliva (FRAS) and the Trolox equivalent antioxidant capacity (TEAC). In addition, other analytes related to metabolism and different tissues and organs, such as lactate dehydrogenase (LDH), creatine kinase (CK), urea and total protein (TP), were included.

## 2. Materials and Methods

### 2.1. Animals

A total of 41 Large White x Landrace pigs (*Sus scrofa domesticus*, 20 males, 21 females) were used in this study, which was carried out at the Veterinary Teaching Farm of the University of Murcia (Guadalupe, Murcia, Spain). Animals were located in groups with a minimum space of 0.65 m^2^ per animal according to Spanish law [22] and an average temperature of 23 ± 2 °C. All animals were in their growing-finishing phase (100 days, 60 kg mean body weight), and were vaccinated against *Mycoplasma hyopneumoniae* and Porcine circovirus type 2 at weaning. Pigs were fed ad libitum with a finishing diet (2.40 Mcal/kg NE and 149.6 g/kg CP) and had free access to water.

This research was approved by the Bioethical Commission of Murcia University (Approval number, CEEA 780/2022; Approval date, 11 March 2022).

### 2.2. Saliva Sampling

**Figure 1:**
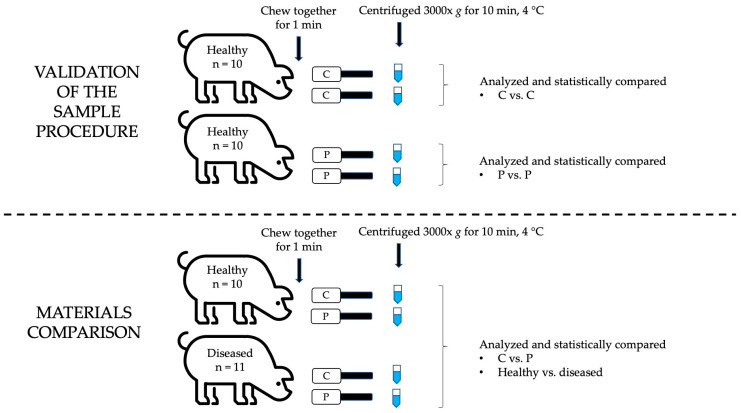
Schematic representation of the experimental procedure. C—cotton roll. P—polypropylene sponge.

The procedures used for the experiments of this report are outlined in Figure 1. Animals were sampled between 09:30 and 10:30 am. Saliva was collected using two different materials: cotton rolls (Salivette^®^, Sarstedt, Aktiengesellschaft and Company, D-51588 Nümbrecht, Germany), with a mean (standard deviation) weight of 529.7 (44.5) mg, and polypropylene sponges (Esponja Marina, La Griega E. Koronis, Madrid, Spain), which were manually cut into 5 × 2 × 2 cm fragments weighing 511.9 (44.8) mg as previously reported [23]. Each collection material was clipped to a flexible thin metal rod. This system allows the pig to chew two devices at the same time by introducing both into the same side of the pig’s mouth. The pigs were allowed to chew the sampling devices for one minute. Then, the absorbent materials were placed into the Salivette^®^ tubes for centrifugation. Tubes were cooled for less than 2 h and immediately centrifuged (3000× *g*, 10 min, 4 °C) once in the laboratory in order to collect the supernatants. The volume of saliva obtained with both materials was quantified and compared. For this, samples were weighed before and after they were centrifuged and the obtained weight in grams was equated to volume in milliliters. Then, samples were stored at −80 °C until the analyses were conducted. Only clean samples were included in the study in order to avoid interference with the analytical methods due to dirtiness.

### 2.3. Validation of the Sampling Procedure Used for the Comparison of the Materials

Two equal pieces of cotton (each attached to a flexible thin metal rod) (cotton–cotton combination) were used in 10 healthy pig’s mouths for one minute in order to obtain two different samples (C1 and C2). Similarly, two equal pieces of polypropylene sponge (sponge—sponge combination) were used for obtaining two saliva samples (P1 and P2) from the other 10 healthy pigs. To evaluate whether this sampling procedure produces samples of similar characteristics, the results obtained for the different analytes in samples C1 vs. C2 and P1 vs. P2 were compared. The hypothesis was that the two samples obtained at the same time and from the same materials would not show significant differences between them in the analytes measured due to the sample collection system. Once it was proven that saliva samples with no significant differences in analytes could be obtained using this double collection system, two different collection materials could be tested at the same time with this procedure.

### 2.4. Comparison between Cotton versus Polypropylene for Saliva Collection

The effect of different sampling materials on the salivary results was evaluated. For this purpose, one device with cotton and another one with polypropylene sponge (cotton–sponge combination), were used simultaneously for sampling a total of 21 pigs. Those animals were subdivided into two different groups. Group 1 (*n* = 10) comprised animals with no clinical signs of disease at farm inspection, so they were considered as healthy. Group 2 (*n* = 11) comprised animals with lameness, which was diagnosed based on the scoring system published by Main and coworkers [24]. An animal was considered lame when it achieved a score ≥1 for their lameness score. Lame animals were housed apart from the healthy pigs in isolation pens, according to Spanish law [25].

### 2.5. Measurements

The measurements in this study included analytes previously measured and validated in porcine saliva by our research group [26,27]. The following analytes were measured.

#### 2.5.1. Stress Biomarkers

Cortisol and oxytocin were measured by in-house immunologic methods using AlphaLISA technology (PerkinElmer, Inc., Hopkinton, MA, USA) with a 96-well fluorometry plate reader (PerkinElmer, Inc., Hopkinton, MA, USA). Both methods had been previously developed and validated for porcine saliva samples.

#### 2.5.2. Immune System and Inflammation Biomarkers

ADA was measured by a kinetic spectrophotometric assay (Adenosine Deaminase assay kit, Diazyme Laboratories, Poway, CA, USA). Mpx was measured by a spectrophotometric method using o-dianisidine as a substrate. Both were measured in an Olympus AU600 biochemistry autoanalyzer (Olympus AU600, Olympus Diagnostica GmbH, Ennis, Ireland). Hp was measured by an in-house assay based on AlphaLISA technology using the fluorometry plate reader.

#### 2.5.3. Sepsis Biomarkers

PCT was measured by an immunologic in-house method using AlphaLISA technology with the fluorometry plate reader.

#### 2.5.4. Redox Biomarkers

CUPRAC was determined based on the reduction of Cu^2+^ to Cu^1+^ by the nonenzymatic antioxidants of the specimen. TEAC was measured by the reduction of 2,20-azino-bis (3 ethylbenzthiazoline-6-sulfonic acid) radicals by the sample. FRAS was determined by the reduction of ferric-tripyridyltriazine (Fe^3+^-TPTZ). All of them were measured in the Olympus AU600 biochemistry autoanalyzer.

#### 2.5.5. Analytes Related to Metabolism and Status of Different Tissues and Organs

CK, LDH and urea were measured by routine spectrophotometric methods from Beckman Coulter. TP was analysed by a spectrophotometric assay (Protein in Urine and CSF, Spinreact, Barcelona, Spain). All the assays showed an imprecision lower than 15% and were linear after serial sample dilution. These analyses were performed in the Olympus AU600 biochemistry autoanalyzer.

### 2.6. Statistical Analysis

All data were previously assessed for normality by the Shapiro–Wilk test, giving a nonparametric distribution. Then, data were expressed as medians (25th–75th percentile) and the statistical analyses were performed by nonparametric methods. The results obtained with a similar material (sponge–sponge and cotton–cotton combinations) were compared by the Wilcoxon test. The results obtained from each pair of different materials (sponge–cotton) were assessed by three different procedures. Firstly, after logarithmic transformation of data, a two-way ANOVAs of repeated measures followed by Sidak’s pairwise comparisons were performed, in which the results obtained were compared considering the different materials and health status of the animals as fixed factors. Secondly, each pair of data was plotted and analyzed by linear regression analysis. Although this approach can ensure a linear relationship between the results obtained with different materials, this does not assume an agreement between the results [28]. Finally, Bland–Altman plots were constructed in which the Y axis shows the difference between two paired measurements and the X axis represents the average of these measurements. This approach quantifies the agreement between two quantitative measurements, as well as establishing the limits for this agreement [29]. The bias between measurements, the 95% confident intervals for this bias and the limits of agreement were calculated according to published protocols [28]. A bias was considered as significant when zero was not included in the 95% confidence interval. Statistical analyses were performed using the GraphPad Prism 8 statistical package (GraphPad Software, San Diego, CA, USA) and the significance was set at *p* < 0.05.

## 3. Results

The results obtained in the validation of the sampling procedure (cotton–cotton and sponge–sponge) are shown in Table 1. No statistically significant differences were found between the results obtained either with the two saliva samples obtained with cotton or with the two samples obtained with the polypropylene sponge.

**Figure 2:**
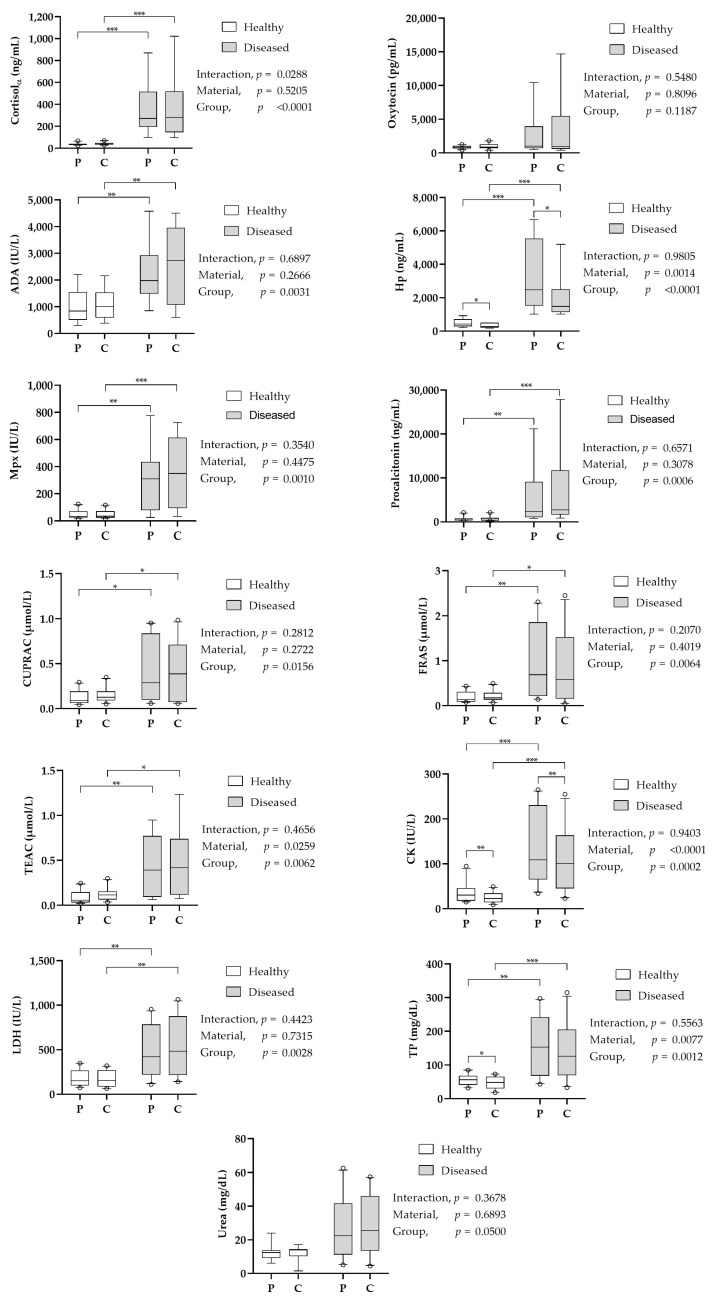
Results of the different salivary analytes in paired saliva samples obtained with polypropylene sponge (P) and cotton roll (C). ADA—adenosine deaminase; CK—creatine kinase; CUPRAC—cupric reducing ability of saliva; FRAS—ferric reducing activity of saliva; Hp—haptoglobin; LDH—lactate dehydrogenase; Mpx—myeloperoxidase; TP—total protein; TEAC—Trolox equivalent antioxidant capacity. Statistical analysis: *—*p* < 0.05; **—*p* < 0.01; ***—*p* < 0.001.

The results obtained with the cotton–polypropylene combination in both healthy and diseased animals are shown in Figure 2. Median (25th–75th percentiles) saliva volumes obtained with the cotton roll and polypropylene sponge were 1.33 (1.11–1.55) mL and 1.67 (1.23–2.09) mL, respectively, being significantly higher (*p* = 0.020) with polypropylene. When the volume of saliva obtained was corrected for the grams of material used for collection, the obtained volumes were 2.50 (2.10–2.93) mL/mg and 3.26 (2.40–4.09) mL/mg of cotton and polypropylene, respectively, being significantly higher (*p* = 0.010) with polypropylene.

The material used for saliva collection had no significant effect in the cases of cortisol, oxytocin, ADA, Mpx, PCT, CUPRAC, FRAS, TEAC, LDH and urea. On the other hand, the material used for saliva collection significantly affected the results of CK, Hp and TP, which yielded lower values in the samples collected with the cotton roll. When healthy and diseased animals were compared, all analytes showed a similar difference between groups with both cotton rolls and polypropylene sponges, as can be observed in Figure 2.

**Figure 3:**
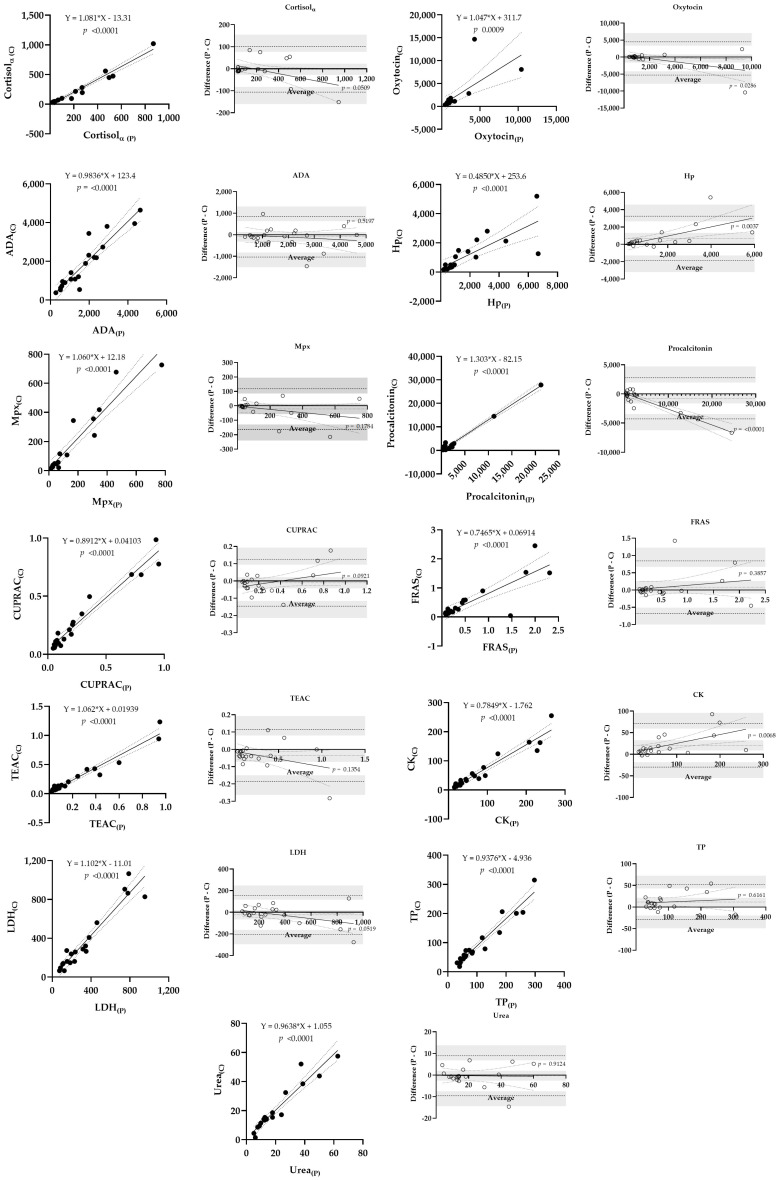
Linear regression and Bland–Altman plots constructed with the results of the paired saliva samples obtained with polypropylene sponge (P) and cotton roll (C). Dotted lines show the 95% limits of agreement. In the Bland–Altman plots, the shaded area indicates the 95% confidence interval of the bias and limits of agreement. ADA—adenosine deaminase; CK—creatine kinase; CUPRAC—cupric reducing ability of saliva; FRAS—ferric reducing activity of saliva; Hp—haptoglobin; LDH—lactate dehydrogenase; Mpx—myeloperoxidase; TP—total protein; TEAC—Trolox equivalent antioxidant capacity. A significant *p* value indicates proportional bias between measurements.

Linear regressions and Bland–Altman plots constructed with the values obtained with the cotton–sponge combination are shown in Figure 3. The regression results showed that the slope was not significantly close to zero in all analytes. Bland–Altman plots showed a bias significantly far from zero (outside the 95% confidence interval) for CK, Hp and TP analyses, with a significant proportional bias for CK and Hp in favor of polypropylene. The rest of the analytes showed a bias within the 95% confidence interval, although proportionality was observed for oxytocin and PCT in favor of cotton.

## 4. Discussion

In this report, the effects of two different materials for saliva collection in pigs, cotton rolls and polypropylene sponges, in the values of salivary biomarkers were evaluated. Both materials have been reported for saliva collection in pigs. Cotton was chosen because it is one of the most common materials used for saliva collection and is also included in commercial devices [30]. In addition, polypropylene sponges have been widely used for the same purpose [23]. Saliva collection was performed with both materials at the same time. When two pieces of the same material were used at the same time for collection (cotton–cotton or sponge–sponge combinations), there were no significant changes in the values of the studied analytes; therefore, the sample procedure designed in our study would be adequate to compare the results of samples obtained at the same time but with two different materials.

The volume of saliva collected by a sampling device can be highly influenced by several factors, such as the collection duration time, the absorbent capacity of the collection device and the amount of saliva available for absorption [31]. It is important to consider this when collecting saliva because those devices that have low saturation thresholds may not estimate reliably salivary flow rate. This could be particularly important for those analytes that are produced and released from the salivary glands because they could be highly influenced by flow rate, decreasing salivary levels as the collection time increases [31]. In our research, the collection time was limited to 1 min, and with those conditions the volume of saliva obtained was between 25 and 30% higher when the polypropylene sponge was used. This indicates that the use of a polypropylene sponge for saliva collection in pigs would be more appropriate when a wide panel of biomarkers is going to be measured and therefore a higher sample volume is needed. To the authors’ knowledge, only one report has been published in which the volume of saliva obtained with different materials was compared. In it, the sample volume was higher with cotton than with the use of synthetic materials [31], although it was performed in humans where the collection device was only kept inside the mouth and not chewed as usually happens with pigs. Moreover, the size of the material devices used could influence these results. In our experimental conditions, it seemed that polypropylene sponge would be more appropriate than cotton to obtain higher sample volumes from pigs.

When the results obtained with the different materials were compared, the analytes cortisol, oxytocin, ADA, Mpx, PCT, CUPRAC, FRAS, TEAC, LDH, and urea did not show statistically significant differences between the saliva collected with cotton rolls or polypropylene sponges, either in healthy or diseased animals. In the case of cortisol, many authors have reported an effect of cotton collection compared with passive drooling [9,10,11,32], although this comparison was not performed in our report. Other reports, in contrast, did not find effects on cortisol measurement due to cotton [14,33]. No previous reports have been found regarding the rest of the analytes we examined. Despite the results observed, caution must be taken regarding some analytes because, for example, in the case of oxytocin, the Bland–Altman plots showed a proportional negative bias for oxytocin when polypropylene was used, indicating that polypropylene could give lower values of oxytocin than cotton when measuring samples with high concentrations. In spite of this, the differences in each of these analytes between healthy and diseased animals were of the same magnitude when the two materials were used. Therefore, such biases may not have any relevance from a clinical point of view, although it is recommended to establish reference values for each material.

On the other hand, there were three analytes (CK, Hp and TP) that showed significant differences in the values obtained with cotton and polypropylene, as well as biases significantly far from zero, indicating significant differences between the samples obtained with each different material. The differences were, in general, equally observed in healthy and diseased animals. From a practical point of view, these results indicate that the collection material could affect the results, considering that the highest values were observed with polypropylene. Therefore, the values in samples obtained with those two different materials should not be compared. These results were confirmed by the Bland–Altman analyses, on which the bias observed for TP was out of the 95% confidence interval and significant proportional biases were observed in CK and Hp assays. Despite the differences observed between the materials, both showed a similar difference between healthy and diseased animals with all tested analytes. Therefore, the use of specific reference values for each collection material should be established for the three analytes (CK, Hp and TP) in order to avoid misinterpretation of the results.

This study indicates that there are some markers that show differences depending on the sampling material, whereas other markers were not affected. Further studies, such as ones taking direct measurements of the analytes in the collection material before and after sampling, should be performed to elucidate whether the reason for the change is the possible presence of the analyte in the material. In addition, to determine which method is better, ideally the samples obtained with the different collection materials should be compared with saliva obtained with passive drool. However, this approach is impractical in pigs unless a previous catheterization of the salivary gland has been performed.

This study has several limitations. Firstly, the study should be replicated using more samples in a larger population of animals. In addition, samples from animals affected by different diseases with different severities should also be included. Furthermore, the study should be also conducted with piglets, so as to evaluate whether the age of the animals could have any influence on their affinity for the collection material. Similarly, the gender should be considered in further studies, because many analytes can differ in their salivary concentrations depending on sex. For this purpose, a larger population of animals of each different sex should be tested. Finally, because this was a preliminary report, a limited panel of measurements was included, so a wider panel of analytes should also be measured in future studies.

## 5. Conclusions

The collection material used to obtain the saliva samples from pigs influences the sample volume, obtaining 25–30% more volume with polypropylene sponges than with cotton rolls. Furthermore, the material can have an influence on the levels of some salivary analytes, with higher values of CK, Hp, and TP obtained in saliva collected with polypropylene. This effect was mainly observed when the saliva contained high values of those analytes, although, in general, the differences detected between healthy and diseased animals in our report were of similar magnitude with both materials. Based on these results, the use of the same collection material type would be desirable when comparing the results obtained from different studies or trials.

## Figures and Tables

**Table 1:** Values of volumes and analytes obtained in cotton devices placed in the same collector clamp (C1 and C2) and sponges placed in the same collector clamp (S1 and S2) in healthy animals. Median (25th–75th percentiles) are expressed.

	Cotton Roll (*n* = 10)		Polypropylene Sponge (*n* = 10)	
	C1	C2	P1	P2
Obtained saliva volume (mL)	1.07 (0.95–1.62)	1.25 (1.15–1.94)	2.00 (1.13–2.48)	1.80 (1.50–2.25)
Stress biomarkers				
Cortisol (ng/mL)	59.8 (48.5–76.4)	57.4 (45.2–70.8)	33.8 (26.3–52.0)	39.6 (18.2–48.8)
Oxytocin (pg/mL)	1031 (886.5–1547)	1214 (880–1441)	848.2 (777.7–1029)	898.6 (749.2–1142)
Inflammation and immunity biomarkers				
ADA (IU/L)	1604 (864.5–2015)	1313 (703.8–1794)	1403 (959.8–2345)	1968 (865.5–2600)
Hp (ng/mL)	344.1 (261.2–682.5)	384.1 (202.0–1062.0)	506.9 (341.9–662.6)	644.7 (373.1–805.5)
Mpx (IU/L)	80.1 (35.6–165.4)	71.1 (30.4–153.1)	55.6 (33.0–104.0)	73.7 (38.7–154.4)
Sepsis biomarkers				
Procalcitonin (ng/mL)	549.0 (355.8–636.8)	419.8 (302.3–578.7)	737.6 (259.6–1626)	921.1 (632.4–1231)
Redox biomarkers				
CUPRAC (µmol/mL)	0.221 (0.141–0.301)	0.214 (0.106–0.252)	0.158 (0.079–0.306)	0.166 (0.087–0.209)
FRAS (µmol/mL)	0.296 (0.145–0.391)	0.332 (0.142–0.370)	0.228 (0.101–0.34)	0.252 (0.115–0.314)
TEAC (µmol/L)	0.167 (0.051–0.237)	0.193 (0.065–0.238)	0.191 (0.058–0.249)	0.196 (0.075–0.286)
Other analytes				
CK (IU/L)	38.1 (19.48–61.05)	37.1 (19.7–59.73)	49.55 (29.23–62.03)	49.75 (33.98–67.33)
LDH (IU/L)	209.3 (106.5–525.0)	253.9 (107.3–463.7)	252.4 (172.4–444.8)	333.8 (142.0–487.3)
TP (mg/dL)	77.20 (46.90–94.48)	80.07 (48.67–96.85)	75.77 (63.71–91.59)	87.75 (68.86–97.21)
Urea (mg/dL)	18.25 (9.73–25.35)	17.55 (9.20–20.93)	12.70 (8.28–25.70)	12.65 (7.85–20.58)

ADA—adenosine deaminase; CK—creatine kinase; CUPRAC—cupric reducing ability of saliva; FRAS—ferric reducing activity of saliva; Hp—haptoglobin; Mpx—myeloperoxidase; LDH—lactate dehydrogenase; TEAC—Trolox equivalent antioxidant capacity; TP—total protein concentration.

## Data Availability

The data presented in this study are available on request from the corresponding author.
